# Diabetic Nephropathy: Novel Molecular Mechanisms and Therapeutic Avenues

**DOI:** 10.1155/2017/3146524

**Published:** 2017-11-22

**Authors:** Sebastian Oltean, Richard Coward, Massimo Collino, Hans Baelde

**Affiliations:** ^1^Institute of Biomedical & Clinical Sciences, University of Exeter Medical School, Exeter EX1 2LU, UK; ^2^Bristol Renal, Translational Health Sciences, Bristol Medical School, Bristol University, Bristol BS8 1TD, UK; ^3^Department of Drug Science and Technology, University of Turin, 10125 Turin, Italy; ^4^Department of Pathology, Leiden University Medical Center, Leiden, Netherlands

Chronic kidney disease (CKD) is a major health problem worldwide. In Europe it is estimated that ~16% of the general population has CKD stages 1–4 and in US the prevalence is similar at ~13%. CKD is associated with increased risk of cardiovascular disease and mortality. It is therefore important to further our understanding regarding the molecular determinants of CKD and find new ways to manipulate them for therapeutic benefit. A large proportion of CKD is due to diabetic nephropathy (DN), one of the microvascular complications of diabetes. In the Western world, DN is the leading cause of end-stage renal disease and kidney replacement therapy (almost half of these patients), an immense burden on healthcare costs. It is estimated that 40–45% of patients with type I diabetes and 30% of patients with type II diabetes have nephropathy.

Multiple molecular mechanisms of DN progression have been described in recent years; however, mechanism-derived treatments for DN are still lacking. This is partly due to poor understanding of the detailed molecular mechanisms underlying diabetic complications. The standard of care is to control glycaemia but most of the time this does not stop the occurrence of the nephropathy.

There is therefore an increasing need to better understand molecular mechanisms of progression in DN and to develop novel treatments that target specifically these mechanisms and are able to slow progression of the underlying CKD in DN.

This special issue is trying to address these goals by publishing both articles that describe novel molecular pathways implicated in DN and novel treatment ideas.

We do hope that the papers here collected may offer the readers an improved knowledge on innovative and original advances in DN pathogenesis and management.

*Sebastian Oltean* *Richard Coward* *Massimo Collino* *Hans Baelde*

